# Improving Indoor Air Quality by Using Sheep Wool Thermal Insulation

**DOI:** 10.3390/ma14092443

**Published:** 2021-05-08

**Authors:** Andreea Hegyi, Cezar Bulacu, Henriette Szilagyi, Adrian-Victor Lăzărescu, Vasile Meiţă, Petrică Vizureanu, Mihaela Sandu

**Affiliations:** 1 NIRD URBAN-INCERC Cluj-Napoca Branch, 117 Calea Floreşti, 400524 Cluj-Napoca, Romania; andreea.hegyi@incerc-cluj.ro (A.H.); henriette.szilagyi@incerc-cluj.ro (H.S.); 2 MINET S.A., 12 Depozitelor Street, 240426 Râmnicu Vâlcea, Romania; cezar.bulacu@minet.ro; 3 NIRD URBAN-INCERC Bucharest, 266 Şoseaua Pantelimon, 021652 Bucharest, Romania; vasile.meita@gmail.com (V.M.); msandu1970@yahoo.com (M.S.); 4 Faculty of Material Science and Engineering, Gheorghe Asachi Technical University of Iaşi, 41 D. Mangeron St., 700050 Iasi, Romania

**Keywords:** sheep wool thermal-insulating mattresses, air quality, water vapor permeability, formaldehyde

## Abstract

Currently, the need to ensure adequate quality of air inside the living space but also the thermal efficiency of buildings is pressing. This paper presents the capacity of sheep wool heat-insulating mattresses to simultaneously provide these needs, cumulatively analyzing efficiency indicators for thermal insulation and indicators of improving air quality. Thus, the values obtained for the coefficient of thermal conductivity, and its resistance to heat transfer, demonstrate the suitability of their use for thermal insulation. The results of the permeability to water vapor characteristics on the sorption/desorption of water, air, demonstrate the ability to control the humidity of the indoor air and the results on the reduction of the concentration of formaldehyde, demonstrating their contribution to the growth of the quality of the air, and to reduce the risk of disease in the population.

## 1. Introduction

Currently, the population living in developed countries spends about 87% of their time inside buildings [1,2,3]. In this context, huge amounts of fuels (wood, oil, coal, natural gas, burned to produce heat during cold weather, or, for the production of electricity used by heating appliances during cold weather or cooling during hot weather) are used worldwide, together with considerable greenhouse gas emissions, to ensure indoor thermal comfort. At the European level, 55–67% of total energy consumption is dedicated to ensure indoor comfort in living spaces, office buildings, or commercial spaces [1,4,5]. According to the communication from the Commission of the European Parliament, the Council, the European Economic and Social Committee, and the Committee of the Regions, COM/2016/051, heating and cooling is the largest energy sector in the EU where 75% of the required energy is provided from fossil fuels and only 18% from renewable sources. In 2012, the report indicated that, 45% of EU heating and cooling energy is used in the residential sector, 37% in industry, and 18% in the service sector, with space heating accounting for more than 80% of energy consumption in colder climates. According to this report, in order to meet heating needs, almost half of EU buildings have individual boilers installed before 1992, with an efficiency of 60% or less. Moreover, at the EU level, the 2016 reporting shows that 22% of individual gas boilers, 34% of electric direct heating appliances, 47% of fuel-oil boilers, and 58% of coal-fired boilers exceeded their technical life, implying low efficiency and high pollutant emissions [6]. Therefore, the current trend is to reduce fuel consumption, to reduce greenhouse emissions, and to ensure the thermal efficiency of buildings through the use of high-performance insulating materials and systems. However, a thermal-insulating “sealing” which only takes into account the total heat transfer is not desirable because a low rate of ventilation will significantly reduce the quality of the air inside [4,7,8] due to the accumulation of moisture, volatile organic compounds gases—one of which, the most dangerous, classified as a 1B carcinogen is formaldehyde [2,9,10,11], allergens, gases, radioactive materials (radon gas), pollutant, inorganic, resulting in a decrease in the quality of life and even make people ill. The sources of these emissions are diverse, some acting continuously, others intermittently or occasionally: food preparation activities (cooking), personal hygiene activities of the population, sanitation of space or things for personal use (e.g., washing clothes, cleaning surfaces with chemicals or their maintenance by lacquering, periodic waxing), smoking, the purchase of new products (emissions of organic compounds of a new element of furniture, for example), even harmless activities intended for relaxation (perfuming space, burning fragrant sticks) and, last but not least, radon emissions from the soil (radon being a gas with a lower density than air, will climb through each crack of the construction, accumulating in ventilated rooms rarely). Moreover, the degradation of an indicator of indoor air quality can induce the degradation of other indicators, for example, the excessive increase in humidity will become a catalyst for the development of microorganisms, which will determine the need for sanitization of the space by chemical treatment of the affected surfaces in order to remove the biological film (treatment carried out with chemicals, biocides), which will bring into the interior a new amount of emissions of volatile organic compounds. The World Health Organization [4,12,13] argues that it is impossible to eliminate all exposure to these pollutants, but sets the risk limits [4,14] in relation to the concentration of the emission of volatile organic compounds expressed as total volatile organic compounds (TVOC) (Table 1). Accordingly, at the European level, two directives limit emissions of volatile organic compounds, as follows: the Solvent Emissions Directive (SED) 1999/13/EC (fully implemented since 2007) and the Products Directive (PD or DECO) 2004/42/EC (which introduces VOC limits for 2010 that relate to specific products and materials (such as paints) containing VOCs).

The symptoms of irritation, discomfort, and physical illness such as headaches, irritation of the eyes, diseases of the respiratory system, allergic reactions, and disorders of the skin, insomnia, difficulty in concentrating, fatigue, and illness, more severe, possibly fatal [4], which may occur not only because of the high concentrations of TVOC in the indoor air, but also to the high content of moisture which can cause mold and mildew whose spores in the air are just as dangerous. These symptoms are currently associated with the “sick building syndrome” [15,16,17,18,19,20]. Therefore, it is necessary and important that, from the point of view of the impact on human health as a result of indoor air quality, at the execution of thermal insulation works, at least three more factors should be analyzed in addition to the thermal performance: water vapor permeability, water adsorption, and the contribution of the thermal-insulating material to the TVOC concentration.

One of the possibilities of achieving a proper thermal insulation without affecting the indoor air quality can be achieved by the use of natural thermal-insulating materials. Therefore, the production of sheep wool thermal-insulation products has a substantial potential as a result of its numerous advantages: it is an abundant renewable raw material resource, it is made from the waste of the wool that cannot be used in the textile industry, the technology of the embodiment is relatively simple and less polluting, the costs of producing and commissioning work are reduced, presents high durability, low flammability, all of the performances needed for a good thermal insulation material [21,22,23,24,25,26]. In terms of the availability of raw material resources, research carried out at the Politecnico di Torino, Department of Architecture and Design [27,28,29] indicated some aspects of the production and processing of sheep products in the Piedmont Region, Italy, which are similar to the situation in Romania. Thus, sheep farms are populated with milk and meat-producing breeds and less with breeds recognized for the fineness of the wool thread. Moreover, for use in the textile industry, the wool is selected and the categories with thick or semi-thick yarn being extremely low in demand, are stored in improper conditions, discarded or burned, thus having a strong pollutant impact. According to the data presented by the National Institute of Statistics, Romania is the fourth country in the EU in terms of the number of sheep and goats and the fourth/fifth place in terms of the number of sheep and goats/100 ha of land, and each sheep shearing operation generates about 2–2.5 kg of wool/sheep’s head, wool that is not taken by the textile industry, becomes polluting waste. According to the United States Department of Agriculture (USDA) Sheep and Lambs Inventory, in 2011, there were 5.5 million sheep in the US [30], statistics indicating an abundance of animals at the level of many areas throughout the planet. As a rule, these thermal-insulating mattresses are placed on the surfaces of walls, ceilings, etc., inside the rooms, in wooden frames mounted on the wall surface, and intended to support the thermal-insulating material, due to the need for protection from the action of water, known as the high-water absorption that wool has in general, and the need to ensure the conditions of keeping the shape and dimensions, since they are non-woven products. This location, however, has substantial advantages over the air quality inside the rooms.

**Figure 1:**
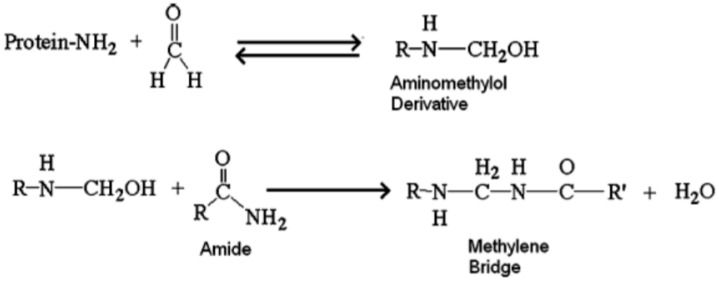
General reaction involving chemisorption of formaldehyde on the wool thread.

Due to the genetic structure, sheep wool yarn has certain peculiarities. It has a natural protein structure, being represented by cuticles whose surface is hydrophobic due to the presence of organic saturated fat, covalently bound to the protein substrate and hydrophilic cortex [21,31] and the medullary canal. Overlapping the cuticles on top of each other determines the roughness and toughness of the thread [21]. The cortex is composed of elongated cells, containing more than 93% by mass, keratin [21,32,33] containing 18 α-amino acids, mainly cysteine, they are responsible for the specific characteristics—gloss, own corrugation, insolubility in water, high resistance to chemicals, low biodegradability, high mechanical strength. The medullary canal is responsible for the characteristic of general hygroscopicity of the wool thread, being known that it absorbs 15–18% of the mass water vapor under normal conditions (20 ± 5 °C, 50–65% RH), but can reach up to 40% under saturated atmosphere conditions (100% RH), absorption that occurs through the expansion of the medullary canal, the diameter of the thread can increase by up to 18% and its length by 1% [21]. According to Downes and Mackay [34], sorption and desorption of moisture under the influence of relative humidity is one of the fundamental properties of wool fibers. Research has shown that, in general, the vapor transfer inside thermal-insulating materials and not only, is influenced by the phenomenon of sorption and the specific resistance to the flow of water vapor, so for two products with equal densities, the water vapor resistance factor, µ, can be much different. On the other hand, it is shown that the action of acidic or basic substances reduces the resistance and increases the castability of the thread, and the increase in temperature and humidity causes the elongation of the wool thread breakage (from 30% for dry thread to 70% for wet thread) because, in accordance with research reported since the 1960s [35,36,37], in the presence of water, a molecular reorganization of the thread. This phenomenon is also supported by other research reported in the literature [38,39], which shows that under conditions of relative humidity of 100%, the wool thread undergoes a process of swelling of 40% on the transverse direction and 1% on the longitudinal direction, with the water molecules entering the protein structure of the thread breaking some protein bonds. The same research indicated that the kinetics of water adsorption on the wool yarn is influenced by the diameter of the yarn, the amount of water adsorbed being inversely proportional to its thickness [39]. Consequently, it can be said that sheep wool is an “smart material”, with a behavior that adapts according to external conditions: it has the ability to absorb water in conditions of high humidity and give it away in conditions of low humidity, thus regulating the humidity of the environment. The behavior of sheep wool in the presence of volatile organic compounds, especially formaldehyde, can be considered a second reason why it can be called a “smart material”. Inside buildings, there are many materials that can emit VOCs, from finishing materials such as paints and varnishes, to treated and processed wood-based composite materials for furniture and finishes, and to elements made of plastics, each of these materials, by their own emissions, variable according to their nature, the time elapsed from installation/use (for example, in the case of a paint, VOC emissions are very high at application, during drying and immediately after drying, decreasing over time), contributing to the accumulation of the VOC (levels indicated in Table 1). Research conducted to date has shown the ability of the wool to adsorb formaldehyde [15,40,41,42,43,44,45,46] and, accordingly, reduce the level of formaldehyde in the inhaled air. This research also showed that the maximum adsorption is achieved at the surrounding temperature of 20 °C, the temperature normally encountered in the living space, and the formaldehyde is absorbed by the wool fibers in one of two ways: physisorption, in which case it is adsorbed in the micropores of the structure of the fibers, and the chemosorbtion, when the formaldehyde forms a chemical bond of stable chains on the side of the amino acids lysine and arginine, and with the groups that the amide of glutamine and asparagine [15] in a chemical reaction, resulting in a pattern (Figure 1). These stable chemical bonds formed will definitively contribute to reducing the concentration of formaldehyde in indoor air. Moreover, the specific character of the formaldehyde thread, which has a keratin structure, allows the chemical binding of a large amount of formaldehyde. Compared to human hair, research has shown that sheep wool has a higher rate of formaldehyde adsorption, thus becoming a “protector” of the population [47]. Studies carried out on the adsorption of formaldehyde, both on strands of hair and sheep wool, have pointed to the fact that, after the binding of formaldehyde to the keratin, the reaction was carried out at the level of the amino group to formaldehyde, which results in the formation of an aminomethylol itself, the process proceeds to a chain reaction that leads to the formation of a solid connection with the methylene bridge, and the release of a molecule of water. To date, no reports have been identified in the literature, indicating the attainment of a degree of formaldehyde saturation of mattress products made of sheep wool. This ability of sheep wool is considered particularly important, especially given that 2–5% of cancer cases reported in the US are attributed to the continuous and prolonged exposure of the population to formaldehyde present in the living environment, schools, offices, or commercial spaces. For extreme situations of high formaldehyde concentration, it is estimated that the risk of cancer increases from 42 cases/million people for a concentration of 10 µg/m^3^, to 252 cases/million people for a concentration of 60 µg/m^3^ [48].

Some studies have shown that wool spontaneously absorbs VOCs in the atmosphere, and thus, it was reported that a capacity reduction of more than 89% of the total concentration of formaldehyde in 3 days after exposure to conditions of 20 °C and 65% RH [49], or the placing of a sample in 150 g of wool into a chamber with a volume of 0.158 m^3^, in which the formaldehyde concentration is 20 ppm, and resulted in the full absorption of the latter within 4 h and, by increasing the formaldehyde concentration to 400 ppm, and in the same condition it was involved in a 35-min drive [50,51].

Moreover, keratin in the wool thread behaves amphoterically, depending on the pH of the medium: at a basic pH, keratin determines an affinity of the thread for negative ions, and at a lower pH, it will determine an affinity of the thread for positive ions [21,52]. This behavior is essential for interaction with the environment, even with pollutants such as oxides, heavy metals, etc. [49]. Thus, some research has shown that under conditions of 2.32 ppm concentration of sulfur dioxide, its absorption capacity by the wool was initially 0.012 mg/gram wool/minute, reaching 0.003 mg/gram wool/minute after 60 min of exposure [50,53]. Research in this direction has been carried out including on the adsorption of toxic products from cigarette smoke, showing the capacity of 40 mg of cigarette smoke/gram of wool [50,54]. Other research has indicated the ability to reduce the concentration of Cu_2_+ by 41% in 1 h, and by 34–53% As_3_+ in 1 h, respectively [55,56].

From the point of view of the intended use and way to produce, in the case of non-woven thermal insulation products, mattresses made of sheep wool, there are both advantages and disadvantages compared to classic thermal insulation products, in particular when comparing to expanded or extruded polystyrene. Thus, classic thermal insulation products are intended for placement, especially on the outside of buildings, on the one hand from the desire not to reduce the internal volume of the rooms, and on the other hand because by design, they significantly reduce the water vapor permeability of the wall. Located indoors will substantially contribute to increasing the humidity of indoor air and, accordingly, increase the likelihood of the development of microorganisms. However, there are situations in which thermal insulation on the outside is not only not the best solution and may even be unfeasible (the case of historical buildings where it is forbidden to modify their exterior appearance) or impossible (constructions with buried walls, not accessible for thermal insulation works). Moreover, the placement inside of thermal insulation materials of the classical type, polystyrene in particular, not only does not allow good ventilation of the walls, but also significantly contributes to the degradation of indoor air quality by the emissions of harmful substances that characterize them. Therefore, the use of a natural thermal insulation sheep wool mattress will cause a small reduction in space, but will also bring major benefits, starting with a feasible thermal insulation solution, up to a significant contribution to air quality (humidity regulation, the possibility of preserving the permeability of the walls, etc.). The reduction of space by thermal insulation inside is a result of the fact that this type of product is placed in wooden frames equal to the thickness of the mattress used, over which sheets of gypsum board or wooden slats are applied. Thus, the resulting surface will be smooth, with sufficient mechanical strength for the application of finishes and for current uses. Therefore, the operation of thermal insulation indoors using mattresses made of sheep wool brings more benefits compared to its main drawback, that of reducing the inner volume of space.

The aim of this paper is to analyze the adsorption processes of water vapor and formaldehyde in the air, simultaneously with the evaluation from the point of view of water vapor permeability of four sheep wool thermal-insulating mattress of Romanian origin. This work will thus contribute to the increase of knowledge in the field of bio-insulating materials used in the construction industry; a sector that is still insufficiently documented. It is therefore considered that it will contribute to the development of this still under-exploited area, to increase the possibilities of using sheep wool that is unproperly used in the textile industry to produce new materials and also to increase the confidence of users.

## 2. Materials and Methods

### 2.1. Materials

**Figure 2:**
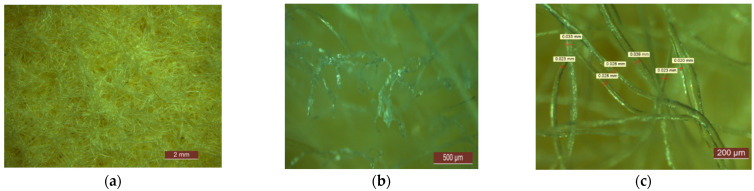
Microscopic analysis of sheep wool thermal-insulating mattresses: (**a**) 1×-overview; (**b**) 5×-identification of the thermal-sealing area; (**c**) 8×-analysis of the thickness of the wool thread.

Four types of products, non-woven type sheep wool mattresses, intended for use as thermal-insulating material in construction, produced within the framework of an experimental research project by MINET SA Râmnicu Vâlcea, Romania, were analyzed. Those were produced by thermal-sealing carding technology of a mixture of 90% sheep wool yarn (thickness between 0.020–0.040 mm) and 10% BICO polymer fibers, characterized by nominal density, thickness, and average real thickness (average result of 10 measurements) according to Table 2. Those were also analyzed microscopically in order to inspect their appearance (Figure 2). For an easier analysis, thermal-insulating mattresses with similar density, within the limits of 30–35 kg/m^3^, were chosen.

### 2.2. Thermal Insulation Efficiency

For the characterization of products from the point of view of thermal insulation efficiency, the coefficient of thermal conductivity, λ_10, ct._ was determined (W/mK) and thermal transfer resistance, R (m^2^ K/W) by means of a Fox 314 conductivity meter, by the hot plate method, at a temperature difference between turntables of 10 °C, according to EN 12667:2002, using specimens of the size 300 × 300 mm^2^, at their natural thickness, after being dried at constant mass in a ventilated oven, the results are presented as the arithmetic mean of five individual measurements for each type of product.

### 2.3. Analysis of the Interaction Phenomenon between Sheep Wool Mattresses and Humidity

Water vapor permeability was quantified by water vapor transmission rate, G (mg/m^2^ h), water vapor resistance, Z (m^2^ h Pa/mg), and by water vapor resistance factor, µ, were calculated according to Equations (1)–(3), where G is the flow of moisture crossing the sample in the time interval Δt, A is the exposed area of the sample, Δp is the atmospheric pressure, δair is the vapor permeability of the air, and d is the thickness of the sample. The tests were performed under laboratory conditions, 65 ± 5% RH and 23 ± 1 °C, using the wet cup method.

g = G/A (mg/m^2^h), (1)

Z = (A × Δp)/G (m^2^hPa/mg), (2)

µ = (δair × Z)/d.(3)

The sorption/desorption capacity of water vapor was quantified by drawing the characteristic curves, by the variation of the sample mass, according to the relative humidity of the air, for 30%, 45%, 60%, 80%, and 95% RH. To stabilize the parameters being followed, the samples were maintained at each RH value for 7 days, after which they were weighed. To ensure repeatability conditions, five determinations were made for each test.

Due to the technology of production of the products, knowing the higher probability of a slight variation in performance, as a result of the specific inhomogeneous structure of the products, it was considered useful to calculate a quadratic mean deviation, as a consequence of a measurement uncertainty, for each set of measurements, the test being conclusively considered if the calculated measurement uncertainty was less than 10% of the average value of the analyzed data.

### 2.4. Influence on Formaldehyde Concentration

**Figure 3:**
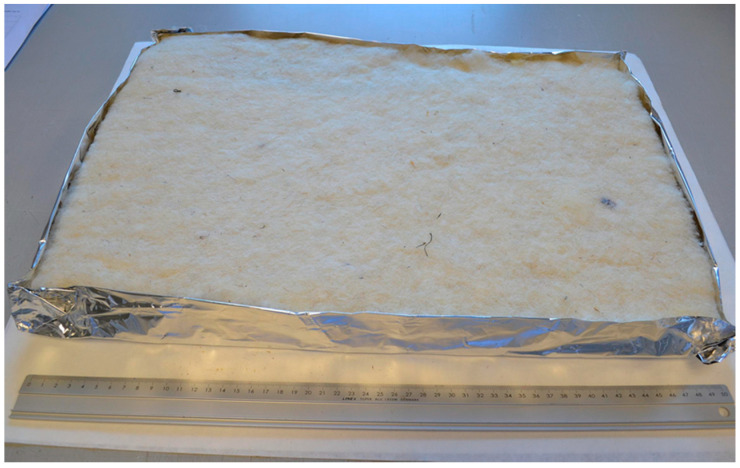
Sheep wool mattress sample.

The sorption/reduction capacity of formaldehyde was finally analyzed, according to ISO 16000-3, for the product that achieved the best performance of those previously presented. The working parameters of the test chamber that complied with the conditions according to EN 16000-9 and EN 16516, are presented in Table 3. The specimen used, with dimensions of 300 × 300 mm^2^ (Figure 3), was covered on the lower face and edges with aluminum foil so that the transfer of formaldehyde was carried out only by the exposed upper face. The testing was carried out over 7 days and the atmospheric concentration of formaldehyde was recorded (day 1, 3, and 7).

Before loading the test chamber, the indoor air was cleaned and checked according to EN 16,516 and ISO 16000-9. During the test, an artificial atmosphere containing a known concentration of formaldehyde was continuously injected into the test chamber to create the basis for the investigation of the reductive properties of the test product, according to Table 3. The presence of aldehydes was tested by air sampling through DNPH-coated silica gel tubes positioned in the exit area of the test chamber after each specified exposure period. The analysis was carried out using an HPLC detector with UV diodes. The absence of formaldehyde and other aldehydes is indicated if the detector’s response to the specific UV wavelength is missing from the chromatogram. The presence and quantification of formaldehyde in the analyzed atmosphere was achieved by comparing specific UV spectra with standard UV spectra.

Formaldehyde consumption, FC (%), was calculated with Equation (4) and sorption flux, F (µg/m^2^∙h), was calculated with Equation (5), where C_in_ = inlet concentration, µg/m^3^; C_out_ = chamber concentration, µg/m^3^; Q_c_ = air flow of chamber = 0.06 m^3^/h and A = area of the test specimen.

FC = (C_in_ − C_out_)/C_in_ × 100 (%), (4)

F = [(C_in_ − C_out_) x Q_c_]/A (µg/m^2^h). (5)

## 3. Results and Discussion

**Figure 4:**
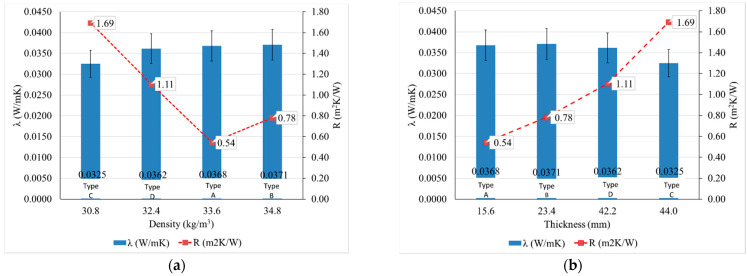
Analysis of thermal insulation performance of thermal insulation sheep wool mattresses: (**a**) influence of material density; (**b**) influence of the thickness of the thermal-insulating mattress (Types A, B, C, D defined according to Table 2).

First of all, in line with the existing literature [1,25,57,58,59], it can be seen the suitability of this material for thermal insulation and the difference between the quantifiable parameters of performance of the thermal insulation: the coefficient of thermal conductivity, λ, is a factor of the material, depending on its density, as the resistance to heat transfer, R, is an indicator of the product, depending on both the specifics of the material and the thickness of the product (Figure 4). It can be said that in the case of tested materials, the coefficient of thermal conductivity, λ, increases with increasing density, and the heat transfer resistance, R, increases with increasing thickness of the product. It can easily be seen that a higher density product does not necessarily provide a lower heat transfer if it does not satisfy a corresponding thickness, so to obtain a high-performance thermal-insulating product, it is necessary to establish an optimal density–thickness balance.

**Figure 5:**
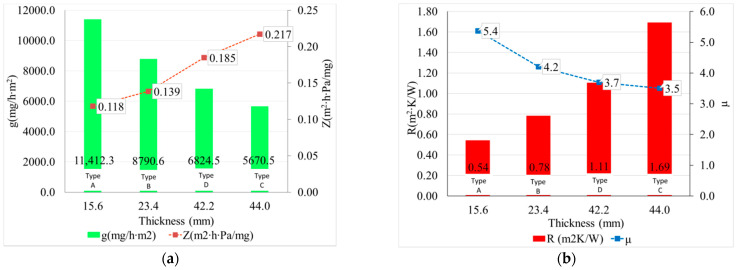
Water vapor permeability quantified by water vapor transmission rate, g (mg/hm^2^), water vapor resistance, Z (m^2^ h Pa/mg), and water vapor resistance factor, µ: (**a**) presented in order of increase in their thickness, respectively (**b**) in correlation with their thermal transfer resistance (Types A, B, C, D defined according to Table 2).

Water vapor permeability performance was analyzed according to the thickness of the thermal-insulating mattresses and in conjunction with the thermal insulation performance quantified as heat transfer resistance and is presented in Figure 5.

It is noted that, regardless of other characteristics of wool mattresses, the water vapor resistance factor, µ, corresponds to values close, in order of magnitude, to values characteristic of other thermal insulation products—mineral wool (µ = 2), glass wool (µ = 1), rigid polyurethane foam (µ = 10), straw or stabilized chipboard plates (µ = 1.5) and are much more permeable than thermal insulation products made of expanded polystyrene (µ = 30–60) or extruded (µ = 150) or open-pore glass (µ = 28), thus ensuring the “breathing” of the walls on which they are used, results that are consistent with specifications from the literature [1]. As expected, the speed of the water vapor transmission decreases with the increase of the thickness of the specimen, but the resistance to vapor-water is less influenced by this increase of the thickness, which might imply that these parameters are affected differently by the characteristics of the test specimen, the first one being dependent upon the characteristic of the product (thickness), and the second one is dependent on the characteristic of the material (density).

**Figure 6:**
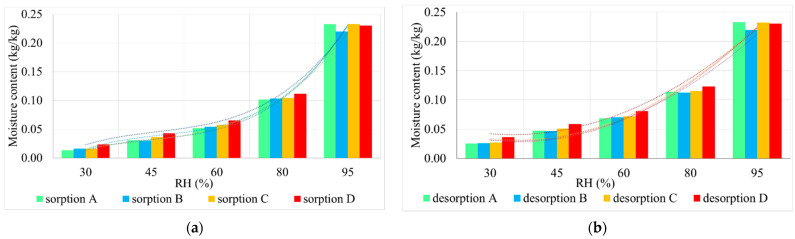
Evolution of the phenomenon of: (**a**) sorption; (**b**) desorption—the sorption/desorption curves obtained by drawing through the points defined as mass variation = f(RH) (Types A, B, C, D defined according to Table 2).

The sorption/desorption capacity of water vapor determined experimentally for thermal-insulating mattresses was achieved by graphical representation of the sorption/desorption curves constructed on the basis of moisture content according to RH (Figure 6). Since these curves are very close, even superimposed in some areas, for better detail, we chose the broken-down graphic representation, Figure 6a for the phenomenon of sorption, and Figure 6b for the phenomenon of desorption, keeping the same proportions to facilitate comparison.

Placing the sorption curve, in all cases, under the desorption curve, signifies that, although permeable to water vapor, the sheep wool thermal-insulating mattresses, under conditions of increased humidity of the environment, are able to retain a certain amount of water, the results being consistent with those reported in the literature [1,25,60]. With the reduction of the humidity of the environment, this water will be released to the environment, but at a lower speed than the retention rate, as demonstrated by the slope of the sorption–desorption curves. As a result of this behavior, it can be appreciated that this type of products contributes not only to the thermal insulation of the interior space but also to the regulation of its humidity. Analyzing the allure of the sorption curves, it can be seen that they are consistent with previous results obtained in the literature [25,34,60,61,62], which indicates that the water absorption of up to 5% of the fiber drying weight is carried out with a higher speed, the water binding strongly, after which, the phenomenon is influenced by the relative humidity of the environment, the speed stabilizing towards the saturation zone, a fact also identified by the analysis of the slope of the sorption curves, respectively, desorption. Considering that the thermal insulation of the building, on the inside can be done for walls, floors, and ceilings, and the volume of indoor air is proportional to their surface area, the literature also points to a high probability of meeting the needs of the improving indoor air quality through the use of this type thermal-insulating material [23,25,27,28,49].

**Figure 7:**
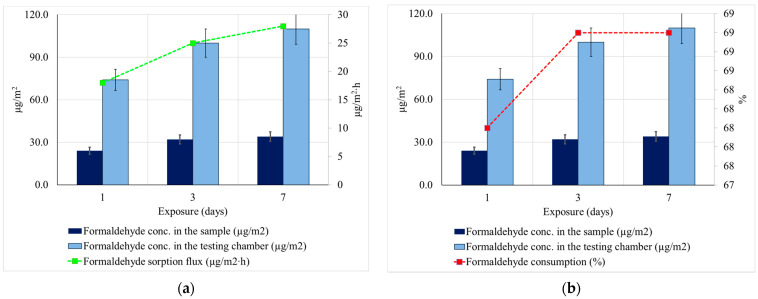
Capacity of the 44 mm thickness sheep wool thermal-insulating mattress on the sorption of formaldehyde. (**a**) Formaldehyde sorption flux (µg/m^2^ h); (**b**) Formaldehyde consumption (%).

As a result of the analysis of the cumulative, it was considered that type-C mattress (30.8 kg/m^3^- density, and 44 mm) meets the simultaneous criteria of performance for thermal insulation, water vapor permeability, and the ability to regulate the humidity of indoor air by sorption/desorption of moisture, according to the need. Thus, for this purpose, the test was performed by the performance of reducing the concentration of formaldehyde in the indoor air, respectively. Binding of this compound by chemisorption, the results being presented in Figure 7.

The results obtained showed that, for equal flows of formaldehyde introduction into the test room, in the situation of the existence of the wool sample, the concentration of formaldehyde recorded in the atmosphere of the enclosure is much lower (24 µg/m^2^ per 1 day, 32 µg/m^2^ per 3 days, respectively, 34 µg/m^2^ per 7 days), compared to the concentration recorded in at 74 µg/m^2^ on 1 day at 110 µg/m^2^ on 7 days). The calculated flow of formaldehyde sorption in the wool test tube was 18 µg/m^2^h at 1 day, 25 µg/m^2^h at 3 days, 28 µg/m^2^h at 7 days, increasing steadily with the duration of exposure and the amount of formaldehyde introduced into the atmosphere inside the test chamber increased, the consumption of formaldehyde by sorption in the wool mattress, calculated, being 68% after the first day of constant, 69%, in the next test period, up to 7 days. Based on these results, the efficiency of the tested thermal-insulating mattress in terms of improving the air quality inside the rooms by reducing the concentration of formaldehyde was demonstrated. Moreover, by keeping the wool sample in the test chamber for another 24 h after the end of the test, under constant conditions and without further introduction of formaldehyde, an amount of 29.5 µg/m^2^ remaining formaldehyde in the sample was determined, which indicates two aspects: the desorption of formaldehyde at 24 h is very low (only 13% of the adsorbed amount and the probability that chemisorption mechanisms that induce chemical bonds, much more stable, than sorption in micropores of the wool thread by mechanisms of a physical nature (physisorption), in accordance with the references in the literature [15,23,49]. According to the literature, this ability to bind formaldehyde also has the advantage that, according to the specialized literature, it becomes, indirectly but effectively, even a passive treatment to increase resistance to attack by microorganisms, insects and their larvae, a concentration of 0.5% formaldehyde is sufficient to provide an annihilation capacity of microorganisms within 5–6 h of exposure [62].

## 4. Conclusions

The aim of this work was to analyze the capacity of sheep wool thermal-insulating mattresses to improve indoor air quality by evaluating the performance of ensuring the water vapor permeability of the thermally insulated surface, regulating humidity by sorption/desorption of atmospheric humidity, and reducing the concentration of formaldehyde, the main toxic substance found in indoor air, under conditions of satisfying the needs of thermal insulation.

Based on the experimental results, it can be said that, according to the literature, sheep wool mattresses have characteristics that successfully fit them into the category of thermal-insulating materials, the coefficient of thermal conductivity being a characteristic property of the material, and the resistance to heat transfer being a characteristic property of the product, that is, influenced by the type of material and its thickness. From the point of view of air humidity, the analyzed products ensure a high degree of comfort by maintaining the “breathability” of the surfaces on which they are applied (walls, ceilings, floors) but also by the ability to regulate humidity through processes of sorption/desorption of atmospheric water. At the same time, these sheep wool mattresses contribute to maintaining the safety and health of the population through the ability to reduce the concentration of formaldehyde.

Tests carried out on the four types of sheep wool mattresses indicated that they have good thermal insulation performance, but, at the same time, they have a beneficial influence on the air in the interior space. Depending on the specific characteristics and the intended use of the products, the use of these materials allows the user to choose the thickness of the mattress, so that both the thermal insulation requirements are met, as well as meeting the limitations imposed by the volume and architecture of the rooms or climatic conditions.

Further studies regarding the possibility of using sheep wool mattresses as thermal insulating materials are focused on statistical analysis regarding the influence of specific production technology on the performance of the product and on identifying optimization opportunities.

## Figures and Tables

**Table 1:** Impact of TVOC [4,14].

TVOC (µg/m^3^)	Health Impact
<200	No irritation or discomfort expected
200–3000	Irritation and discomfort may be possible
3000–25,000	Discomfort expected and headache possible
>25,000	Toxic range where other neurotoxic effects may occur

**Table 2:** Characterization of non-woven type sheep wool thermal-insulating mattresses.

Type	A	B	C	D
Density (kg/m^3^)	33.6	34.8	30.8	32.4
Nominal Thickness (mm)	15 ± 10%	25 ± 10%	45 ± 10%	40 ± 10%
Average Real Thickness (mm)	15.6	23.4	44.0	42.2

**Table 3:** Formaldehyde absorption test parameters.

Parameter	Chamber Volume, V (l)	Air Change Rate, n (h^−1^)	Relative Humidity of Supply Air, RH (%)	Temperature of Supply Air, T (°C)	Average Formaldehyde Injection (µg/m^3^)	Area Specific Ventilation Rate, q (m^3^/m^2^/h)
Value	119	0.5	50 ± 3	23 ± 1	100 ± 20	0.36

## Data Availability

The data presented in this study are available on request from the corresponding authors.
